# Symptomatic Early Congenital Syphilis: A Common but Forgotten Disease

**DOI:** 10.1155/2012/934634

**Published:** 2012-10-09

**Authors:** Machiraju Vasudeva Murali, Cherukuri Nirmala, Jampana Venkateswara Rao

**Affiliations:** Gandhi Medical College and Hospital, Hyderabad 500003, India

## Abstract

Congenital syphilis is a severe, disabling infection often with grave consequences seen in infants. It occurs due to the transmission of the disease from an infected mother to the unborn infant through the placenta. This long forgotten disease continues to affect pregnant women resulting in perinatal morbidity and mortality. The continuing prevalence of this disease reveals the failure of control measures established for its prevention. We put forth a case of symptomatic congenital syphilis presenting with skeletal manifestations at birth, a rare finding in literature. The report stresses upon the importance of implementing the World Health Organization's recommendation that all pregnant women should be screened for syphilis in the first antenatal visit in the first trimester and again in the late pregnancy.

## 1. Introduction

Congenital syphilis is a severe, disabling infection often with grave consequences seen in infants. Syphilis continues to affect pregnant population, in spite of numerous control measures in place established for its prevention. Worldwide, World Health Organization (WHO) estimates that two million pregnant women get infected with syphilis, every year [1]. Without adequate treatment many of them transmit this infection to their offspring, thus increasing the number of reported cases of stillborn, preterm, low birth weight, or congenital infection. 

Congenital syphilis results from transplacental transmission of spirochetes. Approximately 66% of infected infants from congenital syphilis are asymptomatic at the time of birth and are identified only by routine prenatal screening. Untreated syphilis during pregnancy has a transmission rate nearing 100%. Fetal or perinatal death occurs in 40% of affected infants [2]. Clinical signs appear in approximately two-thirds of affected infants from 3rd to 8th week of life and in most cases by three months of age [3]. Primary skeletal involvement is rare [4]. We are reporting one such case of symptomatic congenital syphilis that presented with skeletal manifestations soon after birth, a rare finding in literature [5].

## 2. Case Presentation

A 6 days old full-term, female infant presented with complaints of restricted movements of upper extremities. The infant was born at home through vaginal route and was third in birth order. The infant cried at birth and was exclusively breastfed. On the third day of life, the mother noticed swelling of both the elbow joints. There was no history of trauma, bleeding from any site, bruising, fever, seizures, or altered sensorium.

**Figure 1:**
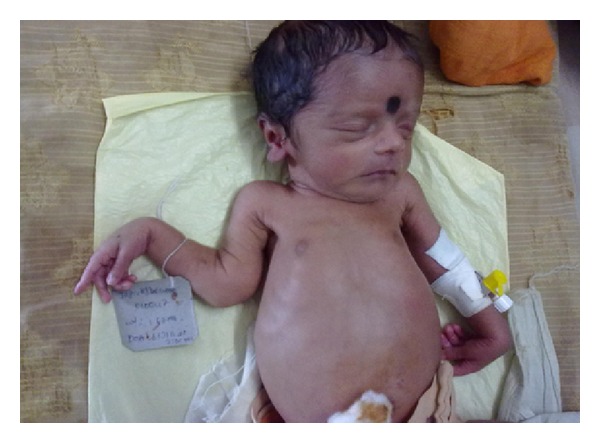
Swelling of both elbow joints.

**Figure 2:**
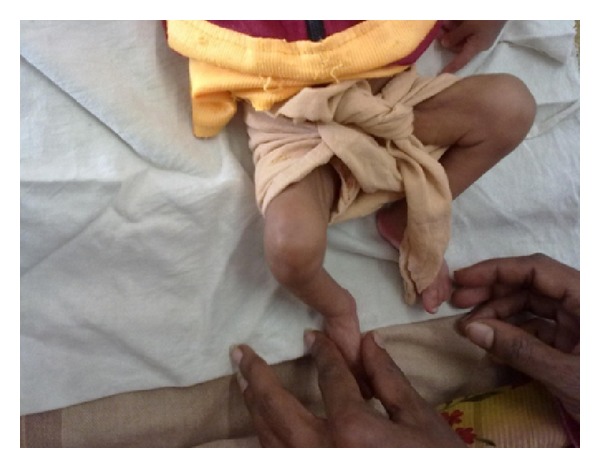
Swelling of right knee joint with no signs of inflammation.

On examination the infant weighed 2.4 kg and was moderately active but irritable. There was mild icterus with no pallor, lymphadenopathy, or rash. Both the upper limbs were in adduction with flexion at elbow joints. There was swelling of both elbow joints (Figure 1) and the right knee joint (Figure 2) along with painful limitation of movements. There was no local rise of temperature or erythema. Marked paucity of spontaneous movements of both upper limbs was observed, and the infant cried on passive movement of the joints (Parrot's pseudoparalysis). The neonate did not have any snuffles or feeding difficulty. Anterior fontanel was flat, and there was no asymmetry of movements or cranial nerve palsy. A differential diagnosis of septic arthritis and traumatic arthritis (Battered Baby Syndrome) was considered. As there was no organomegaly or rash, congenital TORCH infections were not entertained. 

History revealed an uncomplicated antenatal period. The mother was VDRL and HIV negative when screened for in the first trimester. Mother's examination during first visit was noncontributory. The previous two siblings are healthy.

**Figure 3:**
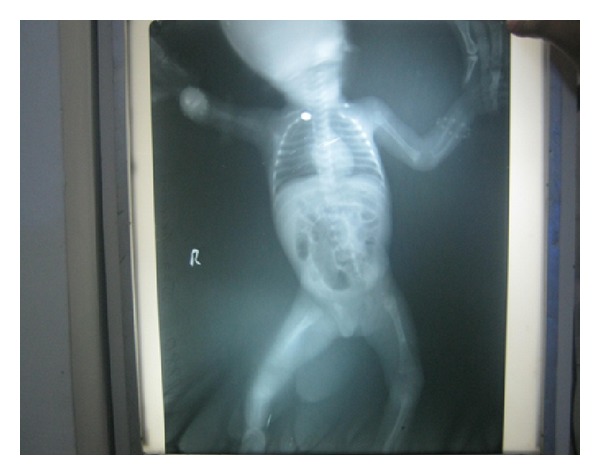
Radiograph showing lower end of humerus (periostitis) and proximal ends of radius and ulna (metaphysitis).

**Figure 4:**
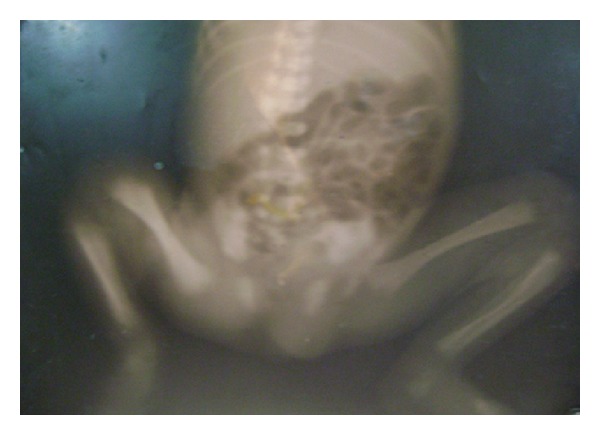
Radiograph of long bones: distal end of femur showing erosions.

**Figure 5:**
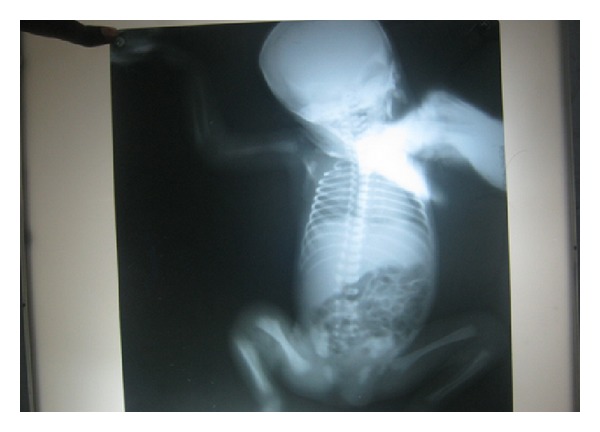
Radiograph of long bones: distal end of femur showing erosions.

Investigations revealed normal blood picture and C-reactive protein (CRP). Blood culture was sterile. Neurosonogram and Ultrasound scan of the abdomen were also normal. An Infantogram revealed osteolytic erosions with indistinct cortical outlines of the ulnar ends of both the humerus. Stippling and erosions of the humeral end of left ulna were also visible (Figure 3). The upper metaphyseal ends of both tibias revealed a typical moth eaten appearance along the medial sides (Wimberger's sign). These changes are classical of congenital syphilis (Figures 4 and 5). 

**Figure 6:**
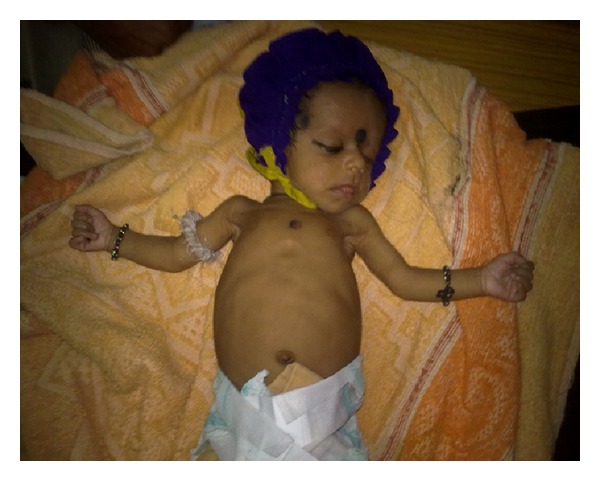
Posttreatment picture showing resolution.

Further, VDRL (Venereal Disease Research Laboratory) test was positive in the baby and the mother with titers being 1 : 2048 and 1 : 128, respectively. Father's VDRL test was negative. CSF VDRL test was positive. Cerebrospinal fluid analysis showed normal sugar and proteins with lymphocytosis. The Treponema pallidum haemagglutination (TPHA) test was positive in the baby, mother, and father. As the father tested negative for the VDRL test but positive for the TPHA test, on further probing it was revealed that the father had undergone treatment for genital ulcers during the first trimester of his wife's most recent pregnancy. Based on the clinical manifestations along with pseudoparalysis, typical radiological findings of osteochondritis, positive VDRL and TPHA tests, a diagnosis of congenital syphilis was made. The infant was managed with crystalline penicillin 50,000 units/kg/dose three times a day for a total of 10 days. The baby became asymptomatic and repeat, VDRL titers were conducted after three months, which were found to be negative. Posttreatment of the radiograph of the long bones showed sclerotic metaphysis with clear bony margins suggestive of resolving metaphysitis. The infant weighed 3.5 kg at followup (Figure 6). Mother was treated by the STD department of the institute as per the protocol.

## 3. Discussion

Congenital syphilis is acquired by an infant from an infected mother by transplacental transmission of *Treponema pallidum* during pregnancy or possibly at birth from contact with maternal lesions [6]. Intrauterine infection with *Treponema pallidum* can result in still birth, hydrops fetalis, or preterm birth, or be asymptomatic at birth. Early form of congenital syphilis is when the clinical manifestations occur before two years of age and late congenital syphilis is when manifestations occur after two years of age [7]. 

General clinical manifestations seen in infants are hepatosplenomegaly, snuffles, lymphadenopathy, mucucutaneous lesions, pneumonia, edema, rash, haemolytic anemia, or thrombocytopenia at birth or within the first 4–8 weeks of age. Primary skeletal involvement, clinically observed as osteochondritis and pseudoparalysis, is rare [8]. The involvement of the skeletal system assumes importance since the frequency and early appearance of roentgenographic changes are of diagnostic value [5]. Typical radiographic findings include periostetis, metaphysitis, and osteitis involving long bones. Syphilitic involvement of bone can be seen along the shaft of the long bones in metaphyseal areas. The radiographic appearance is one of diffuse skeletal involvement that is bilaterally symmetrical. Diagnosis of congenital syphilis is problematic since more than half of all infants are asymptomatic, and signs in symptomatic infants may be subtle and nonspecific [9].

Syphilis is a treatable condition as *T. pallidum* is sensitive to penicillin. VDRL (Venereal Disease Research Laboratory), a nontreponemal test, is commonly used as a screening test for syphilis, which may turn negative after full course of treatment. Treponemal tests like TPHA (Treponema Pallidum Haem-Agglutination) and FTA-Abs (Fluorescent treponemal antibody absorbed) are diagnostic and remain positive even after treatment [10]. 

In the case presented here, the negative results of VDRL for mother (in first trimester) and father misled towards alternate diagnosis. Yet, the confirmatory diagnosis was made by corroborating presented typical radiological findings of osteochondritis and clinical findings like pseudoparalysis. Further, the positive TPHA test of the infant and both parents proved to be conclusive. Father's TPHA test was positive suggestive of syphilis which might have been transmitted to his wife after the first trimester of pregnancy, before he himself underwent treatment for the same. This suggests that in a newborn presenting with joint swellings, one should have a high index of suspicion of syphilis even if the mother is VDRL negative.

 World Health Organization (WHO) recommends that all pregnant women should be screened for syphilis in the first antenatal visit in the first trimester and again in the late pregnancy [11]. We support the recommendation that in addition to the initial testing, a second routine test for syphilis ought to be established early in the third trimester even in low-prevalence areas [12]. Going by this recommendation, had VDRL testing been performed in this mother in late pregnancy, she could have been diagnosed and treated, and the congenital syphilis in the newborn could have been prevented.

Vigilant screening prenatally, at delivery, and an adequate followup are critical to reduce the incidence of congenital syphilis. 

Improved surveillance data and resources are needed for identification and followup of newborns at risk for congenital syphilis [13].
